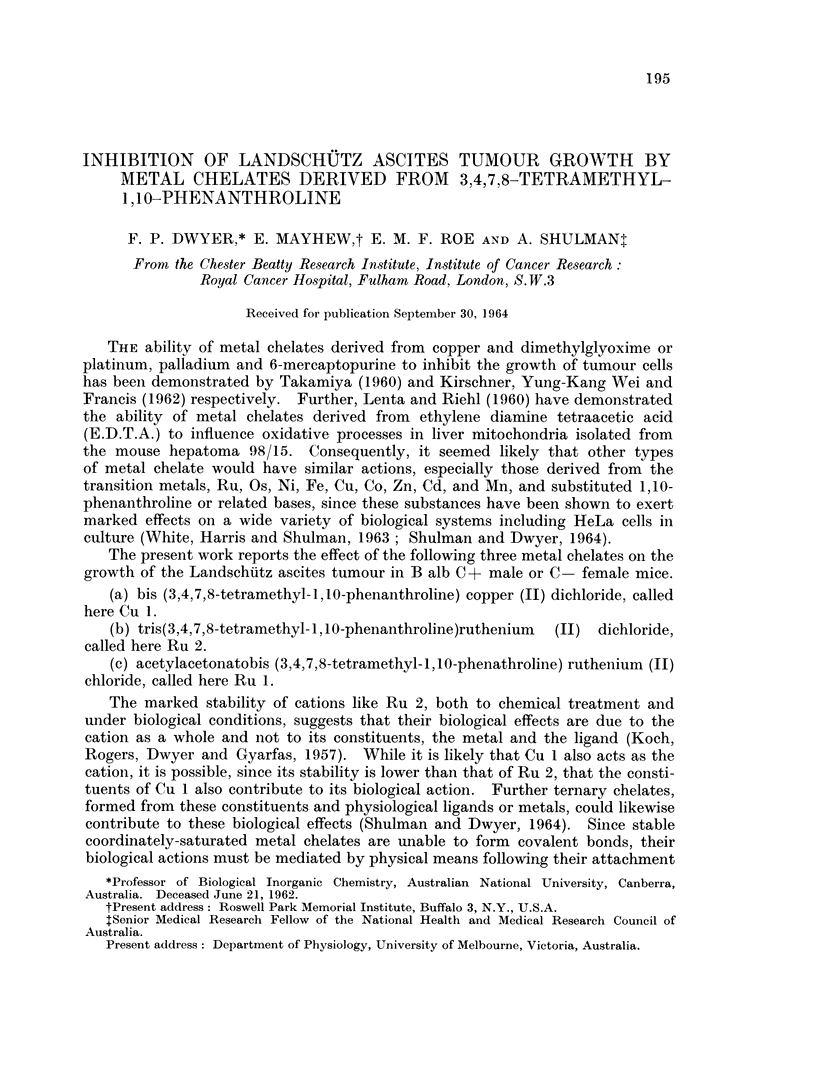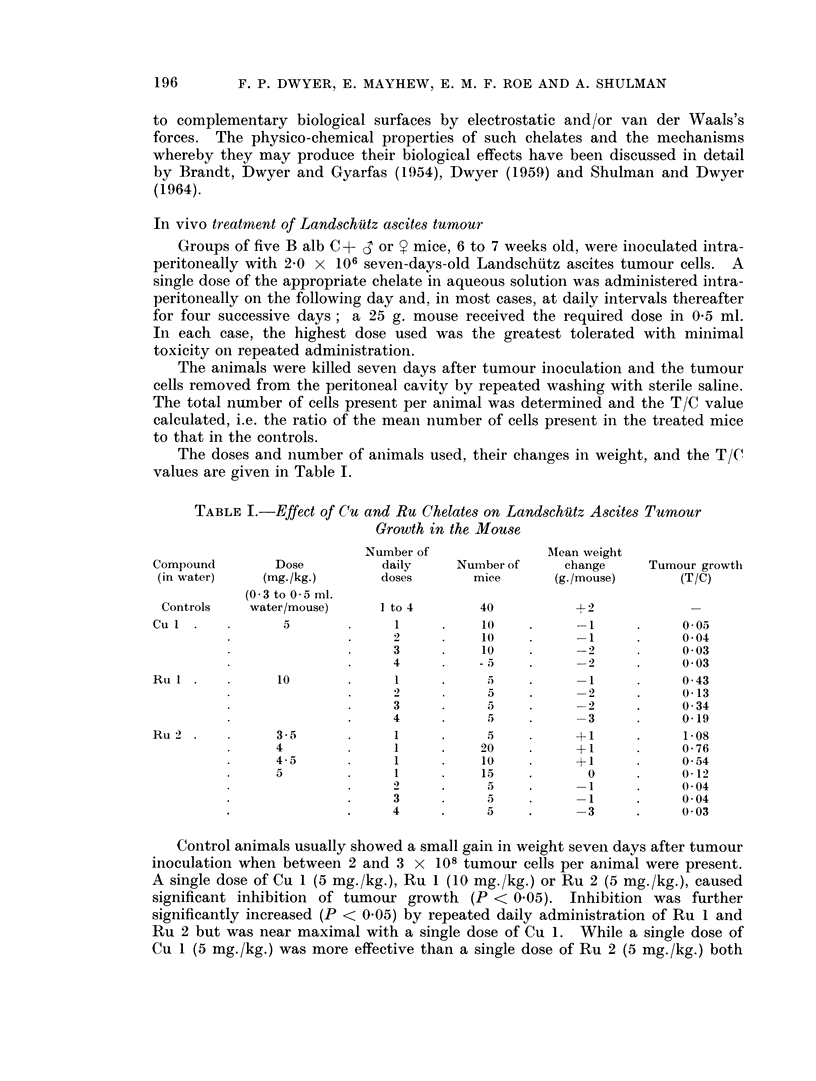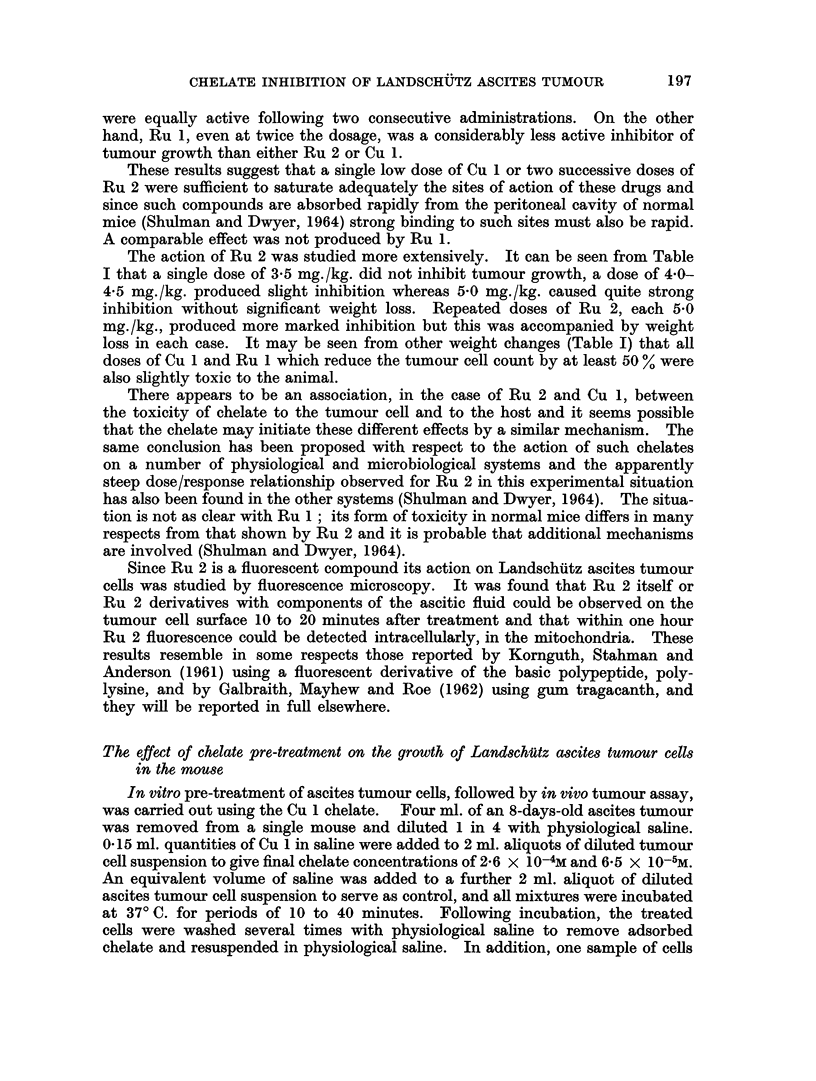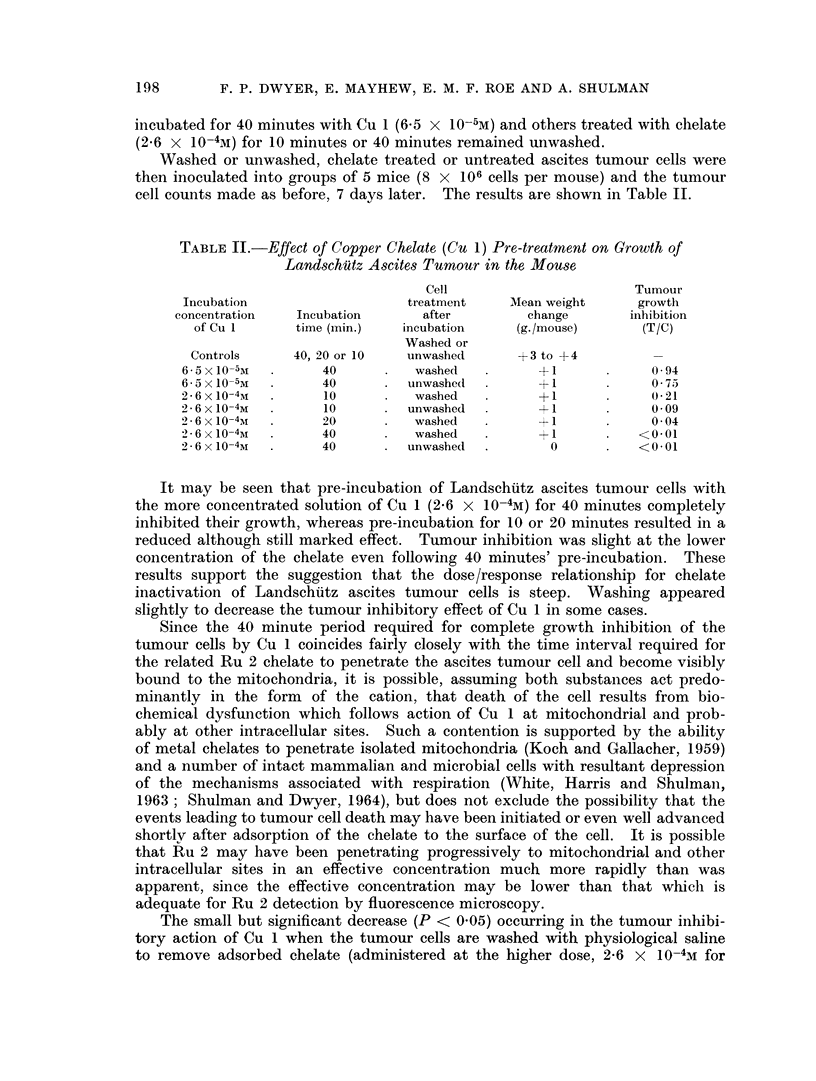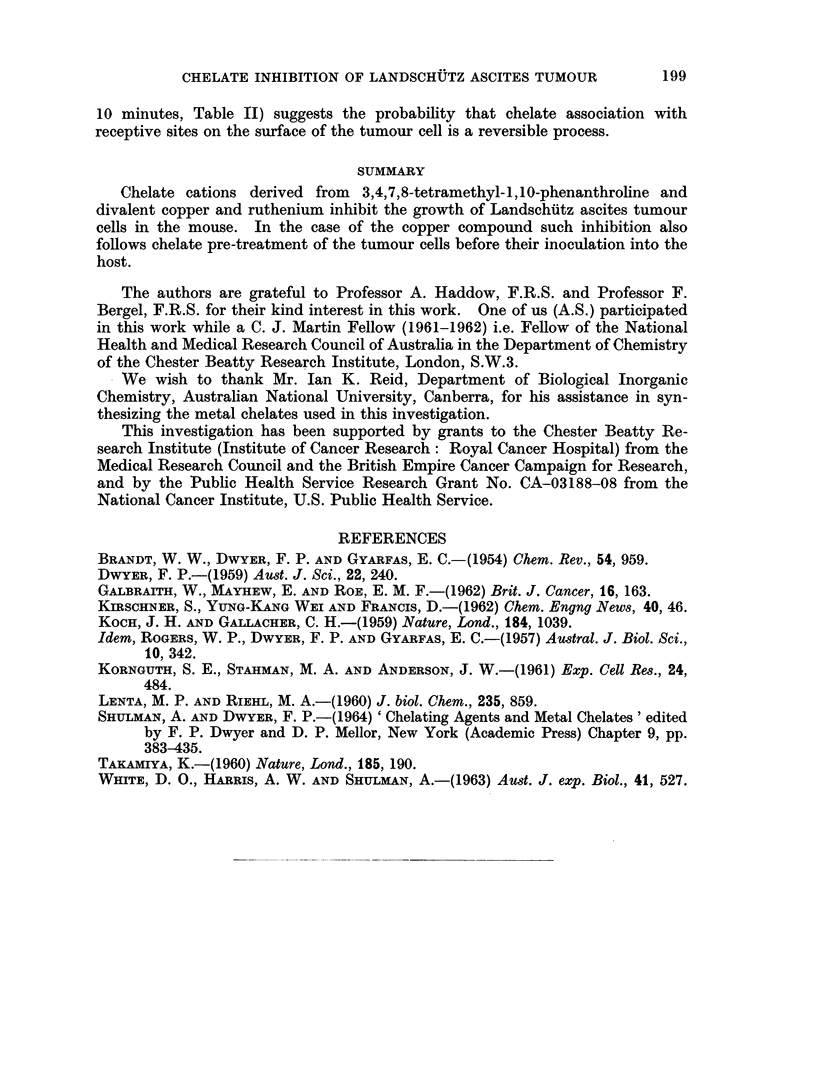# Inhibition of Landschütz Ascites Tumour Growth by Metal Chelates Derived from 3,4,7,8-Tetramethyl-1,10-phenanthroline

**DOI:** 10.1038/bjc.1965.24

**Published:** 1965-03

**Authors:** F. P. Dwyer, E. Mayhew, E. M. F. Roe, A. Shulman


### INHIBITION OF LANDSCHÜTZ ASCITES TUMOUR GROWTH BY METAL CHELATES DERIVED FROM 3,4,7,8-TETRAMETHYL-1,10-PHENANTHROLINE

F. P. DWYER,* E. MAYHEW,† E. M. F. ROE AND A. SHULMAN‡

*From the Chester Beatty Research Institute, Institute of Cancer Research : Royal Cancer Hospital, Fulham Road, London, S.W.3*

Received for publication September 30, 1964

THE ability of metal chelates derived from copper and dimethylglyoxime or platinum, palladium and 6-mercaptopurine to inhibit the growth of tumour cells has been demonstrated by Takamiya (1960) and Kirschner, Yung-Kang Wei and Francis (1962) respectively. Further, Lenta and Riehl (1960) have demonstrated the ability of metal chelates derived from ethylene diamine tetraacetic acid (E.D.T.A.) to influence oxidative processes in liver mitochondria isolated from the mouse hepatoma 98/15. Consequently, it seemed likely that other types of metal chelate would have similar actions, especially those derived from the transition metals, Ru, Os, Ni, Fe, Cu, Co, Zn, Cd, and Mn, and substituted 1,10-phenanthroline or related bases, since these substances have been shown to exert marked effects on a wide variety of biological systems including HeLa cells in culture (White, Harris and Shulman, 1963 ; Shulman and Dwyer, 1964).

The present work reports the effect of the following three metal chelates on the growth of the Landschütz ascites tumour in B alb C+ male or C— female mice.

(a) bis (3,4,7,8-tetramethyl-1,10-phenanthroline) copper (II) dichloride, called here Cu 1.

(b) tris(3,4,7,8-tetramethyl-1,10-phenanthroline)ruthenium (II) dichloride, called here Ru 2.

(c) acetylacetonatobis (3,4,7,8-tetramethyl-1,10-phenathroline) ruthenium (II) chloride, called here Ru 1.

The marked stability of cations like Ru 2, both to chemical treatment and under biological conditions, suggests that their biological effects are due to the cation as a whole and not to its constituents, the metal and the ligand (Koch, Rogers, Dwyer and Gyarfas, 1957). While it is likely that Cu 1 also acts as the cation, it is possible, since its stability is lower than that of Ru 2, that the constituents of Cu 1 also contribute to its biological action. Further ternary chelates, formed from these constituents and physiological ligands or metals, could likewise contribute to these biological effects (Shulman and Dwyer, 1964). Since stable coordinately-saturated metal chelates are unable to form covalent bonds, their biological actions must be mediated by physical means following their attachment

*Professor of Biological Inorganic Chemistry, Australian National University, Canberra, Australia. Deceased June 21, 1962.

†Present address : Roswell Park Memorial Institute, Buffalo 3, N.Y., U.S.A.

‡Senior Medical Research Fellow of the National Health and Medical Research Council of Australia.

Present address : Department of Physiology, University of Melbourne, Victoria, Australia.

to complementary biological surfaces by electrostatic and/or van der Waals's forces. The physico-chemical properties of such chelates and the mechanisms whereby they may produce their biological effects have been discussed in detail by Brandt, Dwyer and Gyarfas (1954), Dwyer (1959) and Shulman and Dwyer (1964).

In vivo *treatment of Landschütz ascites tumour*

Groups of five B alb C+ ♂ or ♀ mice, 6 to 7 weeks old, were inoculated intraperitoneally with 2·0 × $10^6$ seven-days-old Landschütz ascites tumour cells. A single dose of the appropriate chelate in aqueous solution was administered intraperitoneally on the following day and, in most cases, at daily intervals thereafter for four successive days ; a 25 g. mouse received the required dose in 0·5 ml. In each case, the highest dose used was the greatest tolerated with minimal toxicity on repeated administration.

The animals were killed seven days after tumour inoculation and the tumour cells removed from the peritoneal cavity by repeated washing with sterile saline. The total number of cells present per animal was determined and the T/C value calculated, i.e. the ratio of the mean number of cells present in the treated mice to that in the controls.

The doses and number of animals used, their changes in weight, and the T/C values are given in Table I.

TABLE I.—*Effect of Cu and Ru Chelates on Landschütz Ascites Tumour Growth in the Mouse*

| Compound (in water) | Dose (mg./kg.) | Number of daily doses | Number of mice | Mean weight change (g./mouse) | Tumour growth (T/C) |
|---|---|---|---|---|---|
| Controls | (0·3 to 0·5 ml. water/mouse) | 1 to 4 | 40 | +2 | — |
| Cu 1 | 5 | 1 | 10 | −1 | 0·05 |
| | | 2 | 10 | −1 | 0·04 |
| | | 3 | 10 | −2 | 0·03 |
| | | 4 | - 5 | −2 | 0·03 |
| Ru 1 | 10 | 1 | 5 | −1 | 0·43 |
| | | 2 | 5 | −2 | 0·13 |
| | | 3 | 5 | −2 | 0·34 |
| | | 4 | 5 | −3 | 0·19 |
| Ru 2 | 3·5 | 1 | 5 | +1 | 1·08 |
| | 4 | 1 | 20 | +1 | 0·76 |
| | 4·5 | 1 | 10 | +1 | 0·54 |
| | 5 | 1 | 15 | 0 | 0·12 |
| | | 2 | 5 | −1 | 0·04 |
| | | 3 | 5 | −1 | 0·04 |
| | | 4 | 5 | −3 | 0·03 |

Control animals usually showed a small gain in weight seven days after tumour inoculation when between 2 and 3 × $10^8$ tumour cells per animal were present. A single dose of Cu 1 (5 mg./kg.), Ru 1 (10 mg./kg.) or Ru 2 (5 mg./kg.), caused significant inhibition of tumour growth ($P < 0.05$). Inhibition was further significantly increased ($P < 0.05$) by repeated daily administration of Ru 1 and Ru 2 but was near maximal with a single dose of Cu 1. While a single dose of Cu 1 (5 mg./kg.) was more effective than a single dose of Ru 2 (5 mg./kg.) both were equally active following two consecutive administrations. On the other hand, Ru 1, even at twice the dosage, was a considerably less active inhibitor of tumour growth than either Ru 2 or Cu 1.

These results suggest that a single low dose of Cu 1 or two successive doses of Ru 2 were sufficient to saturate adequately the sites of action of these drugs and since such compounds are absorbed rapidly from the peritoneal cavity of normal mice (Shulman and Dwyer, 1964) strong binding to such sites must also be rapid. A comparable effect was not produced by Ru 1.

The action of Ru 2 was studied more extensively. It can be seen from Table I that a single dose of 3·5 mg./kg. did not inhibit tumour growth, a dose of 4·0–4·5 mg./kg. produced slight inhibition whereas 5·0 mg./kg. caused quite strong inhibition without significant weight loss. Repeated doses of Ru 2, each 5·0 mg./kg., produced more marked inhibition but this was accompanied by weight loss in each case. It may be seen from other weight changes (Table I) that all doses of Cu 1 and Ru 1 which reduce the tumour cell count by at least 50 % were also slightly toxic to the animal.

There appears to be an association, in the case of Ru 2 and Cu 1, between the toxicity of chelate to the tumour cell and to the host and it seems possible that the chelate may initiate these different effects by a similar mechanism. The same conclusion has been proposed with respect to the action of such chelates on a number of physiological and microbiological systems and the apparently steep dose/response relationship observed for Ru 2 in this experimental situation has also been found in the other systems (Shulman and Dwyer, 1964). The situation is not as clear with Ru 1 ; its form of toxicity in normal mice differs in many respects from that shown by Ru 2 and it is probable that additional mechanisms are involved (Shulman and Dwyer, 1964).

Since Ru 2 is a fluorescent compound its action on Landschütz ascites tumour cells was studied by fluorescence microscopy. It was found that Ru 2 itself or Ru 2 derivatives with components of the ascitic fluid could be observed on the tumour cell surface 10 to 20 minutes after treatment and that within one hour Ru 2 fluorescence could be detected intracellularly, in the mitochondria. These results resemble in some respects those reported by Kornguth, Stahman and Anderson (1961) using a fluorescent derivative of the basic polypeptide, polylysine, and by Galbraith, Mayhew and Roe (1962) using gum tragacanth, and they will be reported in full elsewhere.

### *The effect of chelate pre-treatment on the growth of Landschütz ascites tumour cells in the mouse*

*In vitro* pre-treatment of ascites tumour cells, followed by *in vivo* tumour assay, was carried out using the Cu 1 chelate. Four ml. of an 8-days-old ascites tumour was removed from a single mouse and diluted 1 in 4 with physiological saline. 0·15 ml. quantities of Cu 1 in saline were added to 2 ml. aliquots of diluted tumour cell suspension to give final chelate concentrations of $2{\cdot}6 \times 10^{-4}$M and $6{\cdot}5 \times 10^{-5}$M. An equivalent volume of saline was added to a further 2 ml. aliquot of diluted ascites tumour cell suspension to serve as control, and all mixtures were incubated at 37° C. for periods of 10 to 40 minutes. Following incubation, the treated cells were washed several times with physiological saline to remove adsorbed chelate and resuspended in physiological saline. In addition, one sample of cells incubated for 40 minutes with Cu 1 ($6 \cdot 5 \times 10^{-5}$M) and others treated with chelate ($2 \cdot 6 \times 10^{-4}$M) for 10 minutes or 40 minutes remained unwashed.

Washed or unwashed, chelate treated or untreated ascites tumour cells were then inoculated into groups of 5 mice ($8 \times 10^{6}$ cells per mouse) and the tumour cell counts made as before, 7 days later. The results are shown in Table II.

TABLE II.—*Effect of Copper Chelate (Cu 1) Pre-treatment on Growth of Landschütz Ascites Tumour in the Mouse*

| Incubation concentration of Cu 1 | Incubation time (min.) | Cell treatment after incubation | Mean weight change (g./mouse) | Tumour growth inhibition (T/C) |
|---|---|---|---|---|
| Controls | 40, 20 or 10 | Washed or unwashed | +3 to +4 | — |
| $6 \cdot 5 \times 10^{-5}$M | 40 | washed | +1 | 0·94 |
| $6 \cdot 5 \times 10^{-5}$M | 40 | unwashed | +1 | 0·75 |
| $2 \cdot 6 \times 10^{-4}$M | 10 | washed | +1 | 0·21 |
| $2 \cdot 6 \times 10^{-4}$M | 10 | unwashed | +1 | 0·09 |
| $2 \cdot 6 \times 10^{-4}$M | 20 | washed | +1 | 0·04 |
| $2 \cdot 6 \times 10^{-4}$M | 40 | washed | +1 | <0·01 |
| $2 \cdot 6 \times 10^{-4}$M | 40 | unwashed | 0 | <0·01 |

It may be seen that pre-incubation of Landschütz ascites tumour cells with the more concentrated solution of Cu 1 ($2 \cdot 6 \times 10^{-4}$M) for 40 minutes completely inhibited their growth, whereas pre-incubation for 10 or 20 minutes resulted in a reduced although still marked effect. Tumour inhibition was slight at the lower concentration of the chelate even following 40 minutes' pre-incubation. These results support the suggestion that the dose/response relationship for chelate inactivation of Landschütz ascites tumour cells is steep. Washing appeared slightly to decrease the tumour inhibitory effect of Cu 1 in some cases.

Since the 40 minute period required for complete growth inhibition of the tumour cells by Cu 1 coincides fairly closely with the time interval required for the related Ru 2 chelate to penetrate the ascites tumour cell and become visibly bound to the mitochondria, it is possible, assuming both substances act predominantly in the form of the cation, that death of the cell results from biochemical dysfunction which follows action of Cu 1 at mitochondrial and probably at other intracellular sites. Such a contention is supported by the ability of metal chelates to penetrate isolated mitochondria (Koch and Gallacher, 1959) and a number of intact mammalian and microbial cells with resultant depression of the mechanisms associated with respiration (White, Harris and Shulman, 1963 ; Shulman and Dwyer, 1964), but does not exclude the possibility that the events leading to tumour cell death may have been initiated or even well advanced shortly after adsorption of the chelate to the surface of the cell. It is possible that Ru 2 may have been penetrating progressively to mitochondrial and other intracellular sites in an effective concentration much more rapidly than was apparent, since the effective concentration may be lower than that which is adequate for Ru 2 detection by fluorescence microscopy.

The small but significant decrease ($P < 0 \cdot 05$) occurring in the tumour inhibitory action of Cu 1 when the tumour cells are washed with physiological saline to remove adsorbed chelate (administered at the higher dose, $2 \cdot 6 \times 10^{-4}$M for

10 minutes, Table II) suggests the probability that chelate association with receptive sites on the surface of the tumour cell is a reversible process.

SUMMARY

Chelate cations derived from 3,4,7,8-tetramethyl-1,10-phenanthroline and divalent copper and ruthenium inhibit the growth of Landschütz ascites tumour cells in the mouse. In the case of the copper compound such inhibition also follows chelate pre-treatment of the tumour cells before their inoculation into the host.

The authors are grateful to Professor A. Haddow, F.R.S. and Professor F. Bergel, F.R.S. for their kind interest in this work. One of us (A.S.) participated in this work while a C. J. Martin Fellow (1961–1962) i.e. Fellow of the National Health and Medical Research Council of Australia in the Department of Chemistry of the Chester Beatty Research Institute, London, S.W.3.

We wish to thank Mr. Ian K. Reid, Department of Biological Inorganic Chemistry, Australian National University, Canberra, for his assistance in synthesizing the metal chelates used in this investigation.

This investigation has been supported by grants to the Chester Beatty Research Institute (Institute of Cancer Research : Royal Cancer Hospital) from the Medical Research Council and the British Empire Cancer Campaign for Research, and by the Public Health Service Research Grant No. CA–03188–08 from the National Cancer Institute, U.S. Public Health Service.

REFERENCES

Brandt, W. W., Dwyer, F. P. and Gyarfas, E. C.—(1954) *Chem. Rev.*, **54**, 959.

Dwyer, F. P.—(1959) *Aust. J. Sci.*, **22**, 240.

Galbraith, W., Mayhew, E. and Roe, E. M. F.—(1962) *Brit. J. Cancer*, **16**, 163.

Kirschner, S., Yung-Kang Wei and Francis, D.—(1962) *Chem. Engng News*, **40**, 46.

Koch, J. H. and Gallacher, C. H.—(1959) *Nature, Lond.*, **184**, 1039.

*Idem*, Rogers, W. P., Dwyer, F. P. and Gyarfas, E. C.—(1957) *Austral. J. Biol. Sci.*, **10**, 342.

Kornguth, S. E., Stahman, M. A. and Anderson, J. W.—(1961) *Exp. Cell Res.*, **24**, 484.

Lenta, M. P. and Riehl, M. A.—(1960) *J. biol. Chem.*, **235**, 859.

Shulman, A. and Dwyer, F. P.—(1964) ‘ Chelating Agents and Metal Chelates ’ edited by F. P. Dwyer and D. P. Mellor, New York (Academic Press) Chapter 9, pp. 383–435.

Takamiya, K.—(1960) *Nature, Lond.*, **185**, 190.

White, D. O., Harris, A. W. and Shulman, A.—(1963) *Aust. J. exp. Biol.*, **41**, 527.